# Iodide of Strontium

**Published:** 1892-09

**Authors:** 


### IODIDE OF STRONTIUM.

(PARAF-JAVAL.)

In this salt of strontium, we have a remedy which advantageously replaces the iodide of potassium in the treatment of *cardiac affections.* Professor See finds that it is, for many reasons, far preferable to the alkaline iodides in these maladies. It does not cause gastralgia or palpitation; it slightly raises vascular pressure, and increases the respiratory functions, and its use does not determine acne or other skin phenomena. It possesses, also, an advantage in the fact that it is speedily eliminated by the kidneys. How far it may finally prove of value in the treatment of syphilis, can only be determined by rigid tests; but the fact that it may be used continuously for months, and in full therapeutic doses without giving rise to gastric disturbances, points to a great future for this salt in the direction cited. In asthma, chronic bronchitis, adenitis, scrofula, arthritis santurnism and hydrargyrism, it has already given remarkable results, as also in the long list of rheumatic and rheumatoid affections.

Dr. Laborde, in a communication to the Society of Biology, remarks that now that strontium salts are generally adopted in practice, he cannot too urgently insist on the necessity of their purity, if further accidents are to be avoided. Recalling his first contributions to the therapeutical uses of strontium, he stated that his physio ogical studies were made with absolutely pure salts, prepared specially by M. Paraf-Javal; these were also used to determine their clinical use, by Drs. G. See, Constantin Paul, Dujardin-Beaumetz, Bucquoy, Ch. Fere and others.

The authenticity of these pure salts, he looked on as an essential condition of success, and he considered it important to bring it to prominent notice.

